# *Mycobacterium Avium* subsp. *Paratuberculosis* Isolates Induce *In Vitro* Granuloma Formation and Show Successful Survival Phenotype, Common Anti-Inflammatory and Antiapoptotic Responses within Ovine Macrophages Regardless of Genotype or Host of Origin

**DOI:** 10.1371/journal.pone.0104238

**Published:** 2014-08-11

**Authors:** Naiara Abendaño, Lyudmila Tyukalova, Jesse F. Barandika, Ana Balseiro, Iker A. Sevilla, Joseba M. Garrido, Ramon A. Juste, Marta Alonso-Hearn

**Affiliations:** 1 Department of Animal Health, Basque Institute for Agricultural Research and Development, NEIKER-Tecnalia, Technological Park of Bizkaia, Bizkaia, Spain; 2 Department of Animal Health, Department of Agriculture of the Regional Government of the Principality of Asturias, SERIDA, Deva, Asturias, Spain; University of Minnesota, United States of America

## Abstract

The analysis of the early macrophage responses, including bacterial growth within macrophages, represents a powerful tool to characterize the virulence of clinical isolates of *Mycobcaterium avium* susbp. paratuberculosis (*Map)*. The present study represents the first assessment of the intracellular behaviour in ovine monocyte-derived macrophages (MDMs) of *Map* isolates representing distinct genotypes (C, S and B), and isolated from cattle, sheep, goat, fallow deer, deer, and wild boar. Intracellular growth and survival of the selected isolates in ovine MDMs was assessed by quantification of CFUs inside of the host cells at 2 h p.i. (day 0) and 7 d p. i. using an automatic liquid culture system (Bactec MGIT 960). Variations in bacterial counts over 7 days from the baseline were small, in a range between 1.63 to 1.05-fold. After 7 d of infection, variations in the estimated log_10_ CFUs between all the tested isolates were not statistically significant. In addition, ovine MDMs exhibited enhanced anti-inflammatory, antiapoptotic and antidestructive responses when infected with two ovine isolates of distinct genotype (C and S) or with two C-type isolates from distinct hosts (cattle and sheep); which correlated with the successful survival of these isolates within ovine MDMs. A second objective was to study, based on an *in vitro* granuloma model, latter stages of the infection by investigating the capacity of two *Map* isolates from cattle and sheep to trigger formation of microgranulomas. Upon 10 d p.i., both *Map* isolates were able to induce the formation of granulomas comparable to the granulomas observed in clinical specimens with respect to the cellular components involved. In summary, our results demonstrated that *Map* isolates from cattle, sheep, goats, deer, fallow-deer and wild boar were able not only to initiate but also to establish a successful infection in ovine macrophages regardless of genotype.

## Introduction

*Mycobacterium avium* subsp. *paratuberculosis* (*Map*) is the causal agent of paratuberculosis or Johne's disease (JD), a chronic inflammatory bowel disease of domesticated ruminants including cattle, sheep, goats and farmed deer, and wildlife worldwide [1], [2], [3]. Paratuberculosis causes major economic losses to the global dairy industry due to lower milk production and reduced slaughter value [4], [5]. *Map* isolates can be classified in two genotypes based on culture characteristics and genome analysis: sheep isolates (also called “S type” or “type I”) and cattle isolates (also called “C type” or “type II”) [6], [7]. Single nucleotide polymorphism (SNP) analysis of the IS*1311* insertion sequence distinguishes three types of strains: S, C and B or “bison” type [8]. Because S-type isolates of *Map* predominate in sheep and C-type in cattle, different host specificities were assumed for both types of isolates.

*Map* is endocytosed by the M cells of the ileal Peyer's patches and subsequently phagocytosed by subepithelial and intraepithelial macrophages [9], [10]. Once inside host macrophages, many phagosomes containing *Map* fail to acquire significant amounts of lysosomal-associated membrane protein (LAMP-1) and to fuse with lysosomes allowing *Map* to persist within infected macrophages [11]. In addition, it has been suggested that *Map* alters the ability of infected macrophages to react to extracellular signals from T cells, particularly through the CD154-CD40 system [12]. *Map*-infected macrophages secrete cytokines and chemokines which contribute to the recruitment of blood and tissue macrophages and T-lymphocytes to the infection site. This organized aggregate of immune host cells around *Map*-infected macrophages is called a granuloma. Within granulomas, activated macrophages differentiate to lipid-loaded macrophages (foamy macrophages), epithelioid cells with large cytoplasms and interdigitated membranes, and/or fuse together to form multinucleated giant cells also called giant Langhans cells [13], [14]. T and B lymphocytes surround the granuloma core, and a tight coat of fibroblasts and collagen closes the structure [15].

The aim of the current study was to to identify differences in virulence between *Map* isolates at two different stages of the infection. Early interaction of *Map* isolates with ovine macrophages was assessed using an ovine monocyte-derived macrophage model. Later stages of the infection, such as the first stages of granuloma formation, were mimicked using an *in vitro* granuloma model. The early interaction of *Map* with subepithelial dome macrophages, its primary host cell, leads to the release of cytokines and chemokines. The differential release of proinflammatory and anti-inflammatory cytokines contributes to the overall cell activation, which may determine whether the pathogen is eradicated or not. Therefore, the analysis of the initial macrophage responses, including bacterial growth within macrophages, represents a powerful tool to rapidly characterize the virulence of clinical isolates of *Map*. By using a bovine macrophage-like cell line (BoMac) and bovine monocyte-derived macrophages (MDMs), we previously observed differences in intracellular growth and persistence in bovine macrophages between strains of *Map* that grouped according to the host of origin [16]. Our results demonstrated that *Map* isolates from goats and sheep persisted within bovine macrophages in lower CFUs than cattle, bison, deer and wild boar strains after 7 days of infection regardless of genotype. A strong correlation between the intracellular multiplication of the tested isolates and patterns of production of host IL-6, TGF-β, MMPL-6, BCL2-1 and IL1-α was observed. Consequently, we suggested that the levels of expression of these proteins might be used to discriminate between isolates of *Map* with differential pathogenicity in bovine macrophages [17]. The intracellular survival within ovine macrophages of *Map* isolates representing distinct genotypes and isolated from a diverse range of hosts has not been fully addressed. Therefore, our first objective was to identify differences in pathogenicity between distinct isolates of *Map* by clarifying which *Map* isolates could potentially initiate disease in an ovine MDM model. For this purpose, we evaluated the capacity of a panel of 10 *Map* isolates representing distinct genotypes to grow and survive within ovine MDMs using an automatic culture system (Bactec MGIT 960). In addition, the expression of several pro- and anti-inflammatory cytokines and genes involved in apoptosis and tissue destruction were tested by qRT-PCR in ovine MDMs infected with the selected *Map* isolates. Because common changes in IL10, TGF-β, and TNFα gene expression were previously observed in human and bovine peripheral blood mononuclear cells (PBMCs), MDMs, and in macrophage-like cell lines infected with *Map* [17], these specific genes were selected for gene expression analysis in ovine MDMs. The expression of the apoptotic inhibitor BCL2-1 and the inhibitor of tissue destruction TIMP-1 was analyzed in ovine MDMs because up-regulation of both genes was previously demonstrated in BoMac cells infected with a bovine isolate of *Map* [16]. Using c-DNA microarrays focused on expressed sequences from a bovine total leukocyte library (BOLT5), significant up-regulation of the antiapoptotic BCL2-A1 gene was also observed in *Map*-infected bovine MDMs relative to uninfected cells [18].

Although MDMs can provide insights into *Map*-host interactions at the very early stages of the infection (7-10 d p. i.), this cellular model is unable to mimic later stages of the infection, such as the first stages of granuloma formation. To address this issue, we have developed a three-dimensional *in vitro* model enabling the formation of *Map-*induced granulomas by incubating infected ovine PBMCs with an extracellular matrix. Since the ability to develop a well-defined granulomatous response was previously shown to correlate with the severity of mycobacterial infections [19], [20], [21], the second objective of our study was to assess whether *in vitro* granuloma formation was induced by two *Map* isolates from cattle and sheep with distinct genotypes.

By comparing the interaction of distinct *Map* isolates with ovine macrophages at two different stages of the infection, we are seeking to identify differences in host specificity and pathogenicity between *Map* isolates and to provide scientific data to support effective control management strategies against ovine paratuberculosis. This information might be very useful in situations where veterinarians and producers have to assess risks and introduce effective management strategies to control paratuberculosis in multispecies livestock operations or on farms where livestock share pastures with wildlife animals potentially infected with *Map.*

## Materials and Methods

### Ethics Statement

Experimental procedures were performed by clinical veterinarians in strict accordance with the recommendations in the Spanish Ethical Guide for the care of animals used for experimental and other scientific purposes (Royal Legislative Decree 53/2013). Blood collection procedure was approved by the Institutional Animal Care and Use Committee (IACUC) of NEIKER-Tecnalia and by the Department of Agriculture, Diputacion Foral de Bizkaia, Spain (Permit N° 14133).

### *Map* Isolates, Bacterial Culture and Preparation of Bacterial Suspensions

Nine *Map* isolates from cattle (*Bos taurus*), sheep (*Ovis aries*), goat (*Capra hircus*), red deer (*Cervus elaphus*), fallow deer (*Dama dama*), and wild boar (*Sus scrofa*) species were selected from the collection of isolates at the Mycobacteria laboratory, NEIKER-Tecnalia, on the basis of varied hosts and genomic profiles as per Sevilla et al., 2007 [22]. These isolates of *Map* were previously recovered from fecal or tissue specimens of domestic or wildlife animal species and maintained as glycerol stocks at −80°C [23], [24]. Aliquots of these glycerol stocks were utilized to directly inoculate all subsequent cultures for use in infection of macrophages. Most of the specimens were collected in several geographic areas of Spain, but three isolates from India, Portugal and The United States were also included in the study. *Map* reference strain K10, a sequenced and laboratory-adapted strain recovered from a clinical case of paratuberculosis, was obtained from the American Type Culture Collection (ATCC) (Manassas, VA). The 10 isolates of *Map* selected for our study were grown in T25 tissue culture flasks at 37±1°C for up to 3 months in 10 ml of Middlebrook 7H9 broth (Difco Laboratories, Detroit, MI) supplemented with 10% (vol/vol) oleic acid-albumin-dextrose-catalase (OADC) (Becton, Dickinson and Company, Franklin Lakes, NJ), 0.05% (wt/vol) Tween-80 (Sigma-Aldrich, St. Louis, MO) and 2 mg L^-1^ of mycobactin J (Allied Monitor Inc., Fayette, MO). Bacterial cells were harvested by centrifugation at 3,000 x rpm for 20 min in a Beckman Coulter Allegra X-12 centrifuge. Bacterial pellets were resuspended in 2 ml of Hank's balanced salt solution (HBSS), and the resultant suspension was passed 20 times through a 27-gauge needle in order to declump cells. The turbidity of the bacterial suspension was adjusted to a McFarland standard of 1 with a Densimat (bioMerieux, Marcy l'Etoile, France). Only the top fraction of the suspension containing dispersed bacteria was used for the infection assays.

### Ovine Monocyte-derived Macrophages (MDMs) Culture

For isolation of ovine mononuclear cells, peripheral blood was collected from the jugular vein of a healthy Latxa sheep older than 48 months. Blood draws were separated by a period of 14 days for red blood cell renewal and a maximum of 1% of the animal's body weight was removed in each blood draw. By monitoring the hematocrit and hemoglobin of the animal, we evaluated whether the animal had sufficiently recovered from a single blood draw. The blood was collected into heparinised Vacutainer tubes (Becton, Dickinson and Company, Sparks, MD), transferred aseptically into sterile glass bottles and diluted 1∶2 in HBSS. Twenty-five millilitres of blood:HBSS were layered over 10 ml of Ficoll-Paque (1,084 g/cm^3^) (GE HealthCare Bio-Sciences, Uppsala, Sweden) in 50-ml centrifuge tubes. Cells were centrifuged at 900× g for 30 min to separate erythrocytes and polymorphonuclear cells from PBMCs. PBMCs were collected from the HBSS-Ficoll-Paque interface and washed with HBSS by centrifugation at 400× g for 10 min. The proportion of purified ovine monocytes was previously estimated in 35.5% of the PBMC fraction obtained by density centrifugation which in itself represents about 70% of the total white blood fraction [25]. The isolated PBMCs were resuspended in Macrophage-serum free medium (Macrophage-SFM) (Invitrogen, Carlsbad, CA) supplemented with 20 mM l-glutamine, 10% heat-inactivated lamb serum (Lonza, Basel, Switzerland), 100 U/ml of penicillin G and 100 µg/ml of streptomycin sulphate. PBMCs were seeded at a density of 1×10^6^ PBMC/ml into 24-well tissue culture plates and incubated for 2 h at 37°C in a 5% CO_2_ incubator. Non-adherent cells were removed by washing twice with HBSS. This step is expected to increase the purity of the purified monocytes. Residual lymphocytes present in the cultures are not expected to influence significantly the experimental results because a normal macrophage-T cell interaction has been shown to be impaired in *Map*-infected macrophages [12]. Adherent cells were incubated for 7 days at 37°C in supplemented Macrophage-SFM to allow differentiation to MDMs prior to infection with *Map.* After 7 days at 37°C most adherent cells were stellate in shape, consistent with macrophage morphology. Incubation of monocytes in Teflon wells or with granulocyte-macrophage colony stimulating factor (GM-CSF) for macrophage maturation are standard protocols for maturing monocytes after Ficoll gradient. However, it was previously suggested that ovine mononuclear blood cells are able to proliferate and differentiate in culture without the addition of growth factors [26].

### Infection of Ovine MDMs with *Map* Isolates from Domestic and Wild Animal Species

Ovine MDMs were inoculated in triplicate with single-cell suspensions of each of the 10 *Map* isolates at a MOI (bacteria:cell) of 10∶1. This level of infection did not alter cell viability over a 1-week assay, as was previously assessed by Trypan blue staining. After a 2 h infection time, the supernatant was removed and the cells were washed twice with HBSS to remove extracellular bacteria. Infected macrophages were lysed at this time point (considered as day 0) or cultured in supplemented Macrophage-SFM medium at 37°C for 7 days (day 7). At each time point, the supernatant was aspirated and infected macrophages were lysed by vigorous pipetting with 0.5 ml of 0.1% Triton X-100 (Sigma-Aldrich) in sterile water for 10 min.

### Viable *Map* Quantification using the Bactec MGIT 960 System

Supplemented Mycobacteria Growth indicator tubes (MGIT) (Becton, Dickinson and Company, Sparks, MD) were inoculated with 0.1 ml of each initial bacterial suspension or with 0.5 ml of the cell lysates for each time point. Each MGIT tube contained 7 ml of modified Middlebrook 7H9 broth base with casein peptone and an oxygen-sensitive fluorescent compound (tris-4,7-diphenyl-1,10-phenathroline ruthenium chloride pentahydrate) embedded in silicone on the bottom of the tube. Each tube was supplemented with 800 µl of an enrichment supplement (BBL MGIT OADC growth supplement) and an antibiotic mixture (BBL MGIT PANTA Antibiotic Mixture) (Becton, Dickinson and Company). The tubes were also supplemented with 2 µg ml^-1^ of mycobactin J. Inoculated vials were incubated at 37±2°C for up to 41 days in the Bactec MGIT 960 instrument (Becton, Dickinson and Company) and were monitored automatically every hour for an increase of fluorescence. The earliest instrumental indication of positivity (i.e., time to detection [TTD]) for each tube was recorded. Any tube that was identified as positive was removed from the instrument, and a sample was tested by PCR to confirm the presence of *Map*. If a tube did not signal positive before 42 days (6 weeks) of incubation, it was removed from the instrument and determined to be negative. The predicted number of bacteria in each positive tube was calculated by using previously generated mathematical formulas which relate TTD (in days) to estimated log_10_ CFUs for each specific *Map* isolate (Table 1) [27].

**Table 1:** IS*1311* PCR-REA types, PFGE profiles and estimations of span, K, and plateau for the quantification of each *M. avium* subsp. *paratuberculosis* isolate in the Bactec MGIT 960 system.

Isolate	Region/Country	Host	PCR-REA	PFGE	Span	*K*	Plateau
K10	United States	Cattle	C	1-1	11.48	0.0503	−1.316
6	Cantabria/Spain	Cattle	C	52-1	16.44	0.0220	−6.080
P38I	Aragon/Spain	Sheep	C	2-1	9.972	0.0211	−0.634
2349/06-1	Portugal	Sheep	S	-	11.82	0.0724	3.222
334	India	Sheep	B	60-1	11.86	0.0298	−0.480
711	Bizkaia/Spain	Goat	C	2-1	17.25	0.0301	−8.208
311	Menorca/Spain	Goat	S	16- 47	9.511	0.0612	3.016
855	Toledo/Spain	Deer	C	68-1	10.95	0.1927	1.317
622/07	Asturias/Spain	Fallow Deer	C	-	21.06	0.0106	−11.25
681	Toledo/Spain	Wild boar	C	2-1	8.755	0.0486	0.760

Growth of all the isolates in the Bactec MGIT 960 system fitted to a one-phase exponential-decay model according to the following equation [log_10_ inoculum size =  span X *e (-K* X TTD) + plateau]. Span is the difference between TTD at time zero and the plateau, *K* is the degree of decay for the log_10_ CFU, and plateau is the value for log_10_ CFU curve flattening.

### Assessment of Uptake, Intracellular Growth and Persistence of *Map* Isolates in Ovine MDMs

Mean estimated log_10_ CFUs in the initial inocula and at days 0 and 7 from three replicate assays were calculated. The percentages of uptake were calculated as the percentages of the inoculated bacteria that were recovered from each cell lysate at day 0. Growth changes between day 0 and day 7 were calculated by dividing the estimated log_10_ CFUs at day 7 by that at day 0 (*n*-fold). The ability of each isolate to persist within host cells is presented as the log_10_ CFUs at day 7.

### RNA isolation, c-DNA Synthesis, and Detection of Several Cytokines and Proteins Involved in Apoptosis or Tissue Destruction by a Two-step Quantitative Reverse-Transcription PCR (qRT-PCR)

Ovine MDMs were inoculated with two ovine isolates of distinct genotype (C and S) or with two C-type isolates from distinct hosts (cattle and sheep) as described above. Uninfected cells were used as controls. At 4, 14 and 24 h p. i., the infected MDMs were washed in 0.5 ml of cold HBSS, mixed with 50 µl of Lysis Solution and incubated at room temperature for five minutes to allow RNA release into the Lysis Solution (PowerSYBR^®^ Green Cells-to-CT™ Kit, Life Technologies, Carlsbad, CA). DNAse I was added to the Lysis Solution to allow genomic DNA degradation at this step. The lysis procedure simultaneously prepares cell lysates for RT-PCR and removes genomic DNA. Next, 5 µl of Stop Solution were mixed into the lysate to inactivate the lysis reagents so that they would not inhibit the reverse transcription (RT) or polymerase chain reactions (PCR). Cell lysates were then reverse transcribed to synthesize cDNA using 20 X RT Enzyme Mix and 2 X SYBR RT Buffer. The reaction mixtures contained 2.5 µl of 20 X RT Enzyme Mix, 25 µl of 2 X SYBR ® RT Buffer, 12.5 µl of Nuclease-free water and 10 µl of the lysate in a 50 µl cDNA synthesis reaction. In order to demonstrate that the template for the PCR was cDNA and not genomic DNA, minus-RT controls containing all the RT components except the 20 X RT Enzyme Mix were prepared for every RNA sample. The reaction mixtures were incubated at 37°C for 60 min and then at 95°C for 5 min to inactivate the RT enzyme. Finally, the synthesized cDNAs were amplified by real-time PCR using the Power SYBR Green PCR Master Mix and the PCR primers set for the target of interest. Real-time qPCR reactions were carried out in triplicate in 25 µl reaction mixtures containing 12.5 µl of Power SYBR Green PCR Master Mix, the optimum concentration of each pair of primers, and 4 µl of cDNA. Using c-DNA synthesized from non-infected cells as template, we had previously determined the concentration of each pair of primers that provided optimal assay performance (i.e., low Ct and maximum ΔRn), but did not produce nonspecific product formation (primer dimmer products) with no-template negative controls (NTC). Primer-dimer products are shorter than the expected amplicons, and thus will have a lower Tm. Real-time qPCR amplifications of cDNAs were accomplished using the ABIPrism 7500 detection system (Applied Biosystem, Carlsbad, CA) under the following conditions: 1 cycle of 95°C for 10 min, 40 cycles of denaturation at 95°C for 15 s, annealing at 60°C for 60 s, and a dissociation curve to measure the specificity of the amplification. Real-time qPCR primers for the amplification of each selected host gene were designed using PrimerExpress 3.0 software and verified for theoretical non-specific annealing with Primer-Blast. Table 2 shows the list of the amplified ovine genes and the corresponding primer sequences. Since the Glyceraldehyde 3-phosphate dehydrogenase (GADPH) gene is constitutively expressed, it was used as the endogenous control gene in the assays. To determine the changes in gene expression (n-fold) or relative quantitation (RQ), the following formula was used: RQ  = 2 -Δ(ΔC_T_) where Δ*C*_*T*_ is *C*_*T*_ (target gene) − *C*_*T*_ (GADPH) and Δ(Δ*C*_*T*_) is Δ*C*_*T*_ (experimental) − Δ*C*_*T*_ (control). Results were expressed as relative quantifications of transcription compared to those of control uninfected cells.

**Table 2:** Genes and primer sequences used in the qRT-PCR assays.

Code Protein		Name	Abbreviation		Primers code/sequence (5′-3)
NM001009806.1	Interleukin 2		IL2	169F/ACAACCCTTGTCTTGCATTGC 170R/CTTGAAGTAGGTGCACCGTTTG	
NM0010093192	TIMP Metallopeptidase inhibitor 1		TIMP-1	173F/TGCTCATCTATCCCCTGCAAA 174R/TGGTCCGTCCACAAGCAA	
NM001009327.1	Interleukin 10		IL10	191F/TTCTTTCAAATGAAGGACCAACG 192R/CCCTTAAAGTCATCCAGCAGAGA	
X55152.1	Tumor necrosis factor, member 2		TNFα- 2	177F/CCATCAGCCGCATTGCA 178R/TTGATGGCAGAGAGGATGTTGA	
NM001009226.1	Mitocondrial protein		BCL2-1	181F/GGGCACTGTGCGTGGAA 182R/TGCGATCCGACTCACCAATA	
NM0010094001	Transforming growth factor, beta 1		TGFβ-1	179F/AAGCGGAAGGGCATCGA 180R/CGAGCCGAAGTTTGGACAAA	
AF030943.1	Glyceraldehyde 3- PO_4_^3^ dehydrogenase		GAPDH	187F/TGCCGCCTGGAGAAACC 188R/CGCCTGCTTCACCACCTT	

### Ovine PBMCs Infection with *Map* Isolates Representing Distinct Genotype and *in vitro* Granuloma Formation

An extracellular matrix (ECM) was prepared according to Kapoor et al. [28] by mixing: 0.8 ml of 3 mg/ml Purecoll collagen solution (Nutacon BV, Leimuden, The Netherlands), 0.1 ml of 10 X Dulbecco's Phosphate buffered saline (DPBS) (Lonza) and 4 µl of 1mg/ml Human Fibronectin (BD Biosciences). The pH of the mixture was adjusted to 7.2–7.6 using sterile 0.1 M NaOH (Sigma). The final volume of the matrix solution was adjusted to 1 ml with sterile water. To prevent gelation, the temperature of the mixture was maintained at 4°C. Fifty microliters of the matrix solution were added to individual wells of a 96-well tissue culture plate. Ovine PBMCs, prepared as described above, were seeded on the ECM at 5 x 10^5^ cells/50 µl ECM/well of a 96-weell plate. *Map* K10 strain or the ovine 2349/06.1 isolate of *Map* were added to each well without touching the surface of the ECM at MOIs (bacteria: cell) of 1∶8, 1∶16 and 1∶33. The ECM was allowed to set by incubating at 37 °C in a 5% CO_2_ incubator for 2 h. The volume of each well was adjusted to 200 µl by the addition of RPMI +20% FBS. Samples were incubated at 37°C in a 5% CO_2_ incubator for 10 days. The media was changed on day 5. Cellular aggregation was observed under an inverted phase-contrast microscope (Olympus IX81) equipped with a Nikon DS-Fi1 digital camera. Images were edited using Fiji/Image J Software (v 1.48). *In vitro* generated aggregates were counted at 5 and 10 days p. i.

### Harvesting Aggregates for Histology

After 10 days of incubation, the medium was carefully removed from each well and replaced with 0.2 ml of 10% neutral buffered formalin. Plates were incubated overnight at 37°C in a 5% CO_2_ incubator overnight. Next day, the formalin was removed and 0.2 ml of hematoxylin diluted 1∶1 in phosphate-buffered saline (PBS) were added to each well. After 10 min of incubation at room temperature, the stain was removed and 0.2 ml of 2% LE-2 agarose (Lonza) heated at 42–45°C was added to each well. Plates were placed at 4°C for 10–15 min to allow agarose to solidify. Agarose plugs containing cellular aggregates were removed from each well and stored in six-well dishes in 70% ethanol before processing for histopathology.

### Paraffin Embedding, Sectioning and Histological Staining

A drop of eosin was applied to the agarose plugs containing aggregates. Each plug was wrapped in tissue wipers (VWR, Radnor, PA, US), placed in a marked cassette and processed on a tissue processor Shandon Citadel 2000 (Fisher Scientific Co., Pittsburgh, PA, US) for 17 h. Each plug was embedded in paraffin wax, and sectioned at 4 µm on a microtome Leica RM2035 (Leica Mycrosystems, Barcelona, Spain). Sections were mounted on treated microscope slides (Fisher Scientific Co) and stained with hematoxylin and eosin (HE) and Ziehl–Neelsen (ZN) stain for acid-fast bacteria. Aggregates were observed under a microscope Olympus BX51 equipped with an Olympus U-CMAD3 digital camera.

### Statistical Analysis

Estimated log10 CFUs in the initial inoculums and 0 and 7 days after infection of ovine MDMs were compared with the General Lineal Model (GLM) procedure of the SAS statistical package version 9 (SAS Institute Inc., Cary, NC). In the analysis; time p.i. (inoculum and days 0 and 7), genotype (C, S and B), and host of origin (cattle, sheep, goat, deer, wild-boar, and fallow-deer) were the independent variables. Cytokine production at 4, 14 and 24 h p. i. was compared with the GLM procedure of the SAS Software. In the analysis; host (cattle and sheep), and time (4, 14 and 24 h p.i.) were the main effects. Numbers of in vitro generated granulomas were compared by one-way analysis of variance (ANOVA) with the Tukey-Kramer multiple-comparison post-test (GraphPad Software, San Diego, CA). In all analyses, differences were considered significant when P values were <0.05.

## Results

### Uptake, Growth and Persistence of *Map* Isolates from Cattle, Goat, Sheep, Deer, Fallow Deer and Wild Boar in Ovine MDMs

Ovine MDMs were infected in triplicate with a panel of 10 *Map* isolates. Isolate code, country of isolation, host of origin, and genotype for each isolate are summarized in Table 1. The log_10_ CFUs present in the initial inocula and after 2 h (day 0) or 7 days (day 7) of infection were estimated for each isolate in the Bactec MGIT 960 system and the corresponding results are presented in Table 3. When the individual means of all the isolates were compared, variations between the estimated log_10_ CFUs in the initial inocula, and at days 0 and 7 p.i. were not statistically significant. When we compared time effect on the overall means, no significant differences among the means of the estimated log_10_ CFUs in the initial inocula and at day 7 were obtained (*P* = 0.3902). In contrast, significant differences between the mean log_10_ CFUs in the inocula and at day 0 were observed for all the strains, which indicated that not all bacteria in the initial inocula was successfully internalized (*P*<0.0001). As shown in table 3, the percentages of uptake in ovine MDMs were estimated in a range between 52% and 87% of the initial inocula depending on the isolate.

**Table 3:** Entry and intracellular growth of Map isolates and the reference strain K10 in ovine MDMs.

Isolate	Host-IS1311 PCR/REA type	Entry (%) a	log_10_ CFU (± SD) b		n-Fold d	*P* value
			Day 0 c	Day 7		
K10	Cattle-C	65.32	4.88 (±2.59)	7.99 (±0.35)	1.63	0.0058 e
681	Wild board- C	73.13	5.33 (±0.99)	8.01 (±0.20)	1.50	0.1901
6	Cattle-C	67.36	5.86 (±2.73)	7.89 (±1.50)	1.34	0.4752
334	Sheep-B	75.01	6.05(±3.28)	7.64 (±3.47)	1.26	0.9942
622/07	Fallow deer-C	79.56	7.48 (±1.66)	8.82 (±0.19)	1.17	0.9998
711P	Goat-C	74.91	5.59 (±0.80)	6.17 (±1.10)	1.10	1.0000
P381	Sheep-C	86.39	7.81 (±1.36)	8.44(±0.22)	1.08	1.0000
311	Goat-S	77.00	7.75 (±2.44)	8.19 (±2.44)	1.05	1.0000
2349/06-1	Sheep-S	51.93	4.93 (±0.05)	4.90 (±0.24)	0.99	1.0000
855	Deer-C	74.45	5.86(±0.73)	5.77 (±0.86)	0.98	1.0000

a Uptake was calculated as the percentage of the inoculated bacteria that was recovered from each cell lysate at day 0.

b Values shown are means of three repeated experiments ± standard deviations (SD).

c Day 0 = 2 h post infection.

d Growth changes (n-fold) were calculated by dividing the number of log10 CFU at day 7 by that at day 0 for each Map isolate.

e Indicates a significant change between day 0 and day 7 (P<0.05).

When time post-infection was considered as the main effect, significant differences between the overall means of the estimated log_10_ CFUs at days 0 and 7 were observed suggesting that variations in log_10_ CFUs occurred during the 7-day incubation period (*P*<0.0001). The intracellular growth exhibited for each isolate between days 0 and 7 in ovine MDMs is individually represented in Table 3 as the calculated fold change. Of the ten isolates, seven isolates (681, 6, 334, 622/07, 711P, P381, 311) and the K10 reference strain were observed to increase in number the initial bacterial concentration (n-Fold >1). However, these variations in bacterial counts over 7 days from the baseline were small, in a range between 1.63 to 1.05-fold, with K10 reference strain proliferating more rapidly than the other tested isolates. Statistical analysis of the data indicated that only the K10 reference strain exhibited significantly increased bacterial counts after 7 days of infection when compared with baseline (*P* = 0.0058). Although the isolates from sheep (2349/06-1) and deer (855) were observed to minimally decrease in log_10_ CFUs over 7 days from baseline (n-Fold <1), this variation was not statistically significant (*P* = 1.000). The ability of each isolate to persist within ovine MDMs is presented as the mean log_10_ CFUs at day 7 in Table 3. After 7 days of infection, variations in the estimated log_10_ CFUs at day 7 between all the tested isolates were not statistically significant.

### Gene Expression in Ovine MDMs Infected with Two C-type Isolates from Distinct Hosts or with Two Ovine Isolates of *Map* with Distinct Genotypes

**Figure 1:**
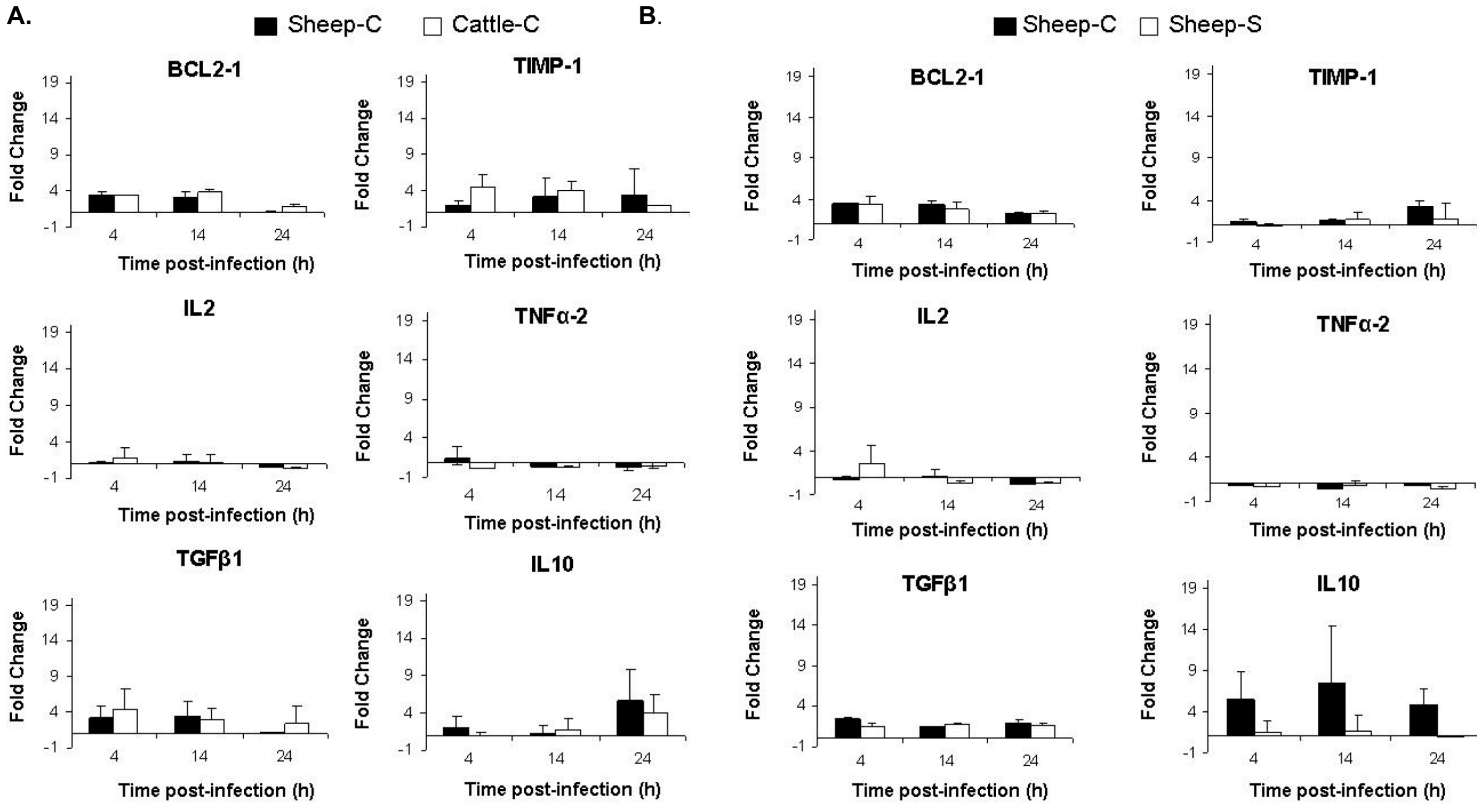
Expression of cytokines and proteins involved in inhibition of apoptosis or tissue destruction in ovine MDMs infected with bovine and ovine isolates of Map. Ovine MDMs were infected with (A) C-type Map isolates from cattle (K10 strain) and sheep (p38I isolate), or with (B) two ovine isolates with C- (p38I isolate) or S-genotype (2349/06-1 isolate). At 4, 14 and 24 h p.i. gene expression was assessed by qRT-PCR. Isolates are identified in the figure by their corresponding host of origin and IS1311 PCR-REA type (C or S). Bars represent the average results of two independent infection experiments (± SD). No statistically significant differences in the expression of the indicated genes between the tested isolates were observed.

In order to determine whether the bacteria's host of origin affects gene expression in *Map*-infected MDMs, two C-type isolates from cattle and sheep were selected for gene expression analysis. The same test was run separately with two ovine isolates with distinct genotypes (C or S) to assess whether the bacteria's genotype would significantly change gene expression in ovine MDMs. Mean fold changes in gene expression between infected and non-infected cells were determined through real-time qRT-PCR analysis and are shown in figures 1A and 1B, respectively. Variations in the expression levels of several cytokines (IL10, TGFβ-1, IL2 and TNFα-2), the anti-apoptotic gene BCL2-1, and the tissue destruction inhibitory gene TIMP-1 in ovine MDMs infected with the two C-type isolates from cattle and sheep or with the two ovine isolates with distinct genotypes (C or S) were not statistically significant. Although from the Figure 1B it looks like that there are variations between the IL10 expression levels in ovine MDM infected with C- and S-type ovine isolates, statistical analysis of the data indicated that these variations were not statistically significant at 4 (*P* = 0.2568), 14 (*P* = 0.3697) and 24 h p.i. (*P* = 0.0914).When the expression of each gene was compared individually at the three time points assessed, ovine MDMs infected with C-type isolates from sheep and cattle for 4 and 14 h p.i. showed significantly increased levels of expression of the apoptotic inhibitor BCL2-1 when compared with the expression of this gene at 24 h p.i.. In general, up-regulation of the anti-inflammatory cytokines IL10 and TGFβ-1, and downregulation of the proinflammatory cytokines IL2 and TNFα-2 were observed in ovine MDMs infected with the selected *Map* isolates. The observed increased expression of the antiapoptotic BCL2-1 gene and of the tissue destruction inhibitory gene TIMP-1 might cause low levels of apoptosis and cellular destruction and allow *Map* persistence in the infected MDMs. In summary, ovine isolates of distinct genotype (C and S) and C-type isolates from sheep or cattle induced anti-inflammatory, antiapoptotic and antidestructive responses in ovine MDMs.

### Infection of Ovine PBMCs with Two *Map* Isolates from Cattle and Sheep with Distinct Genotypes (C and S) Resulted in the Formation of Three-dimensional Microgranulomas

**Figure 2:**
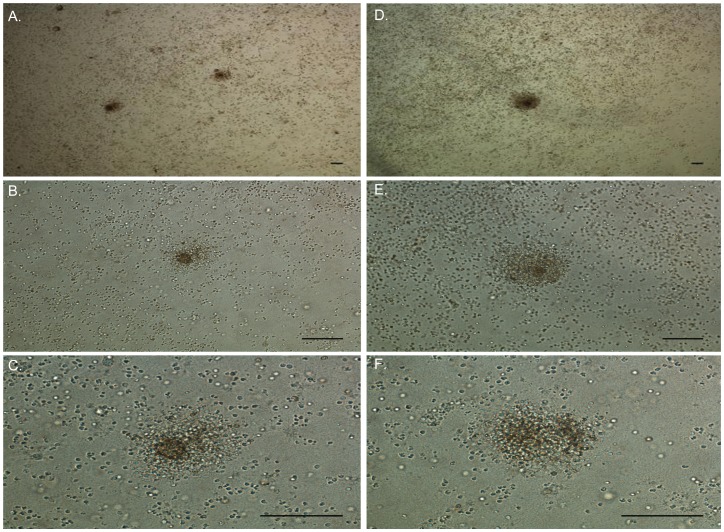
Phase contrast images showing the presence of day-10 granuloma-like aggregates after the infection of ovine PBMCs with bovine and ovine *Map* isolates. Ovine PBMCs (5 x 10^5^) seeded on an extracellular matrix were infected with the bovine K-10 reference strain (A, B and C) or with an ovine isolate of *Map* (2349/06-1) (D, E and F) at MOI (bacteria:cells) of 1:8. Magnification in A and D  =  4X, in B and E  =  20X and in C and F  =  40X. Bars  =  300 µm.

Ovine PBMCs seeded on a collagen matrix were infected with the K10 *Map* reference strain or with the ovine isolate (2349/06-1) at three different MOIs (bacteria: cell) (1∶8, 1∶16, and 1∶33). Previous studies demonstrated that infection of PBMCs with *M. tuberculosis* and *M. bovis* at a MOI 1∶1 or lower resulted in the formation of microgranulomas [28], [29]. Cellular recruitment around the bacteria was followed by light microscopy over a 10-day period. The experiment was repeated tree times with a conserved time course of around 3–5 days for cellular aggregation of lymphocytes around infected macrophages and 5–10 days for the formation of a multilayer, microscopic, granuloma-like aggregate. Both *Map* isolates formed rounded granuloma-like aggregates by day 10 as shown in Figure 2. In control, uninfected PBMCs from the same healthy donor, the formation of aggregates was not observed indicating that aggregation occurs only in response to *Map* infection (data not shown).

**Figure 3:**
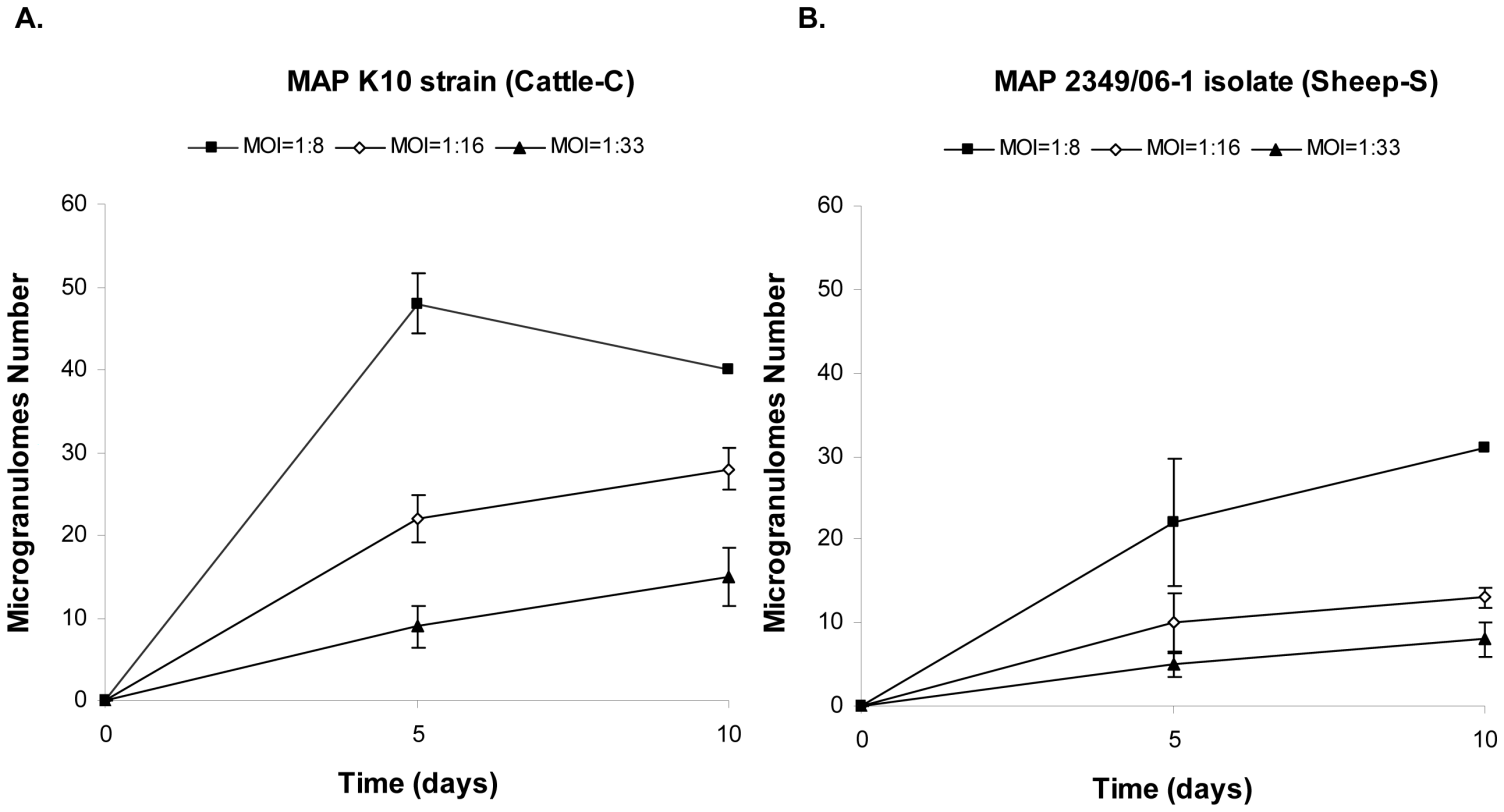
Number of microgranulomes generated *in vitro* 5 and 10 days after infection of ovine PBMCs with (A) the K10 reference strain and with (B) the ovine 2349/06-1 isolate. Aggregate numbers were estimated under a light microscope. The mean aggregate number estimated by triplicate is shown for each MOI (1:8, 1:16 and 1:33) ± SD.

The number of granuloma-like aggregates formed in response to the infection with both *Map* isolates at 5 and 10 days post-infection is shown in Figure 3. Statistical analysis of the data showed significant variations between the number of aggregates generated after 5 days of infection with the K10 reference strain and the 2349/06 isolate at the highest MOI (1∶8), with the K10 strain inducing the formation of more aggregates than the ovine isolate (*P*<0.01). However, after 10 days of infection the number of aggregates formed by both strains was not significantly different at any of the 3 assessed MOIs. The number of aggregates generated by both strains at the highest and lowest MOI (1∶8 and 1∶33) was significantly different at 10 days p. i. Significant differences in the number of aggregates triggered by both strains between days 5 and 10 were not observed which suggested that most of the granulomas were formed during the first five days of the infection with both isolates. Uninfected cells showed no granuloma formation.

**Figure 4:**
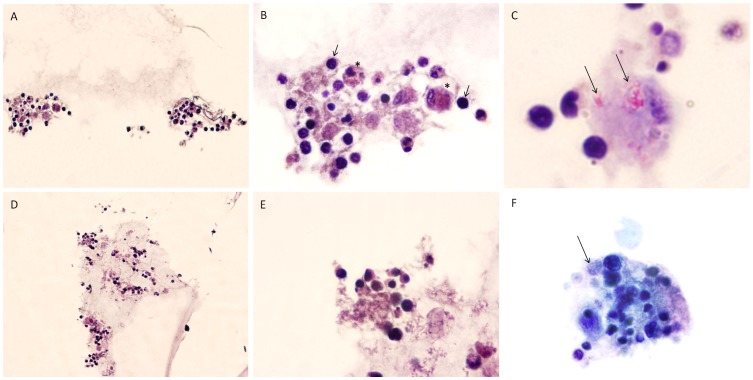
Morphological characterization of the cell populations recruited within *in vitro-* ovine granulomas. Primary ovine PBMCs (5x10^5^) were seeded on an extracellular matrix and subsequently infected with the K10 reference strain (A, B and C) or with an ovine isolate of *Map* (2349/06-1) (D, E and F) at MOI (Bacteria:cells) 1:8. At 10 days p.i., the granuloma-like aggregates were harvested, processed for histopathology and stained with HE (A, B, D and E) and ZN stains (C and F). Original magnification in A and D  =  200X and in B, C, E and F  =  1000X. As shown in image B, macrophages (asterisk) and lymphocytes (arrows) were present in the granulomas. In images C and F, acid-fast bacilli (arrows) were observed within macrophages by ZN staining.

After 10 days of culture, the morphological characterization of the granuloma-like aggregates was confirmed by histological staining. Figure 4 shows the morphological characterization of the aggregates formed by primary ovine PBMCs infected with the K10 *Map* reference strain and with the ovine isolate of *Map* (2349/06-1) at MOI 1∶8. As shown in figures 4B and 4E, granulomas exhibited aggregation of lymphocytes around infected macrophages. When granuloma sections were stained with ZN, *Map* cells could be observed residing within the granulomas.

## Discussion

The identification of *Map* isolates with differential virulence may assist in further elucidating the pathogenesis of paratuberculosis and in the design of better strategies for controlling this infection. The analysis of the initial macrophage responses, including bacterial growth and survival within macrophages, represents a powerful tool to rapidly characterize the virulence of clinical isolates of *Map*. In the current study, we examined the intracellular growth and survival within an ovine MDM model of a panel of *Map* isolates representing distinct genotypes and isolated from cattle, sheep, goats, deer, fallow deer, and wild boar. Woo et al, previously demonstrated that following ingestion by bovine MDMs the number of viable *Map* cells increased during the first 4 days and then declined between days 4 and 8 after infection, as determined by a radiometric method [30]. In accordance with these results, we previously assessed intracellular growth and survival of our panel of *Map* isolates in a bovine macrophage-cell line (BoMac) and in bovine MDMs after 7 days of infection. To ensure consistency across *in vitro* models and to provide enough time for bacteria to grow, growth and survival of *Map* isolates within ovine macrophages was evaluated on day 7. Growth changes between days 0 and 7 were calculated by dividing the estimated log_10_ CFUs at day 7 by that at day 0 (*n*-fold). Of the ten isolates, seven isolates (681, 6, 334, 622/07, 711P, P381, 311) and the K10 reference strain were observed to increase in number the initial bacterial concentration (n-Fold >1). However, these variations in bacterial counts over 7 days from the baseline were small, in a range between 1.63 to 1.05-fold. Although the isolates from sheep (2349/06-1) and deer (855) were observed to minimally decrease in log_10_ CFUs over 7 days from baseline (n-Fold <1), this variation was not statistically significant (*P* = 1.000). Statistical analysis of the data indicated that only the K10 reference strain exhibited significantly increased bacterial counts after 7 days of infection when compared with baseline (*P* = 0.0058). The K10 is a laboratory-adapted strain while the other strains are recently isolated, low passage *Map* isolates. Different levels of aggregation or clumping between *Map* strains can affect the intramacrophage growth of each strain. However, we used a low bacillary inoculum which minimizes mycobacterial clumping during initial infection and more closely mimics conditions *in vivo*, where small numbers of *Map* cells can establish infection.

All the isolates, including the K10 strain, were able to survive in equivalent log_10_ CFUs within ovine MDMs after 7 days of infection. Therefore, an association between a specific host of origin and intracellular survival of the tested isolates in ovine MDMs was not observed. Consequently, we can hypothesize that the conditions encountered by the tested *Map* isolates within macrophages of their respective hosts did not differentially alter the phenotype of the bacteria and their subsequent persistence within ovine MDMs. In contrast, we previously showed that type S and type C isolates from sheep and goats showed a significant attenuated phenotype in a macrophage-like cell line of bovine origin (BoMac) after 7 days of infection, when compared with type C isolates form cattle, deer, fallow deer, wild boar and bison [16]. These observed variations between isolates in bovine macrophages grouped according to the host from which the isolates were isolated and were not associated to the genotype of the isolate. Similarly, strains of environmental Mycobacteria from fish and humans including strains of *Mycobacterium avium*, *Mycobacterium peregrinum*, *Mycobacterium chelonae*, and *Mycobacterium salmoniphilum*, had different abilities to grow within macrophages lines from humans, mice and carp; which grouped according to the host from which the isolates were isolated [31].

Our results suggested that sheep might be susceptible to infection with *Map* isolates not only from sheep and cattle but also from goats, deer, fallow deer and wild boar as well. Therefore, the importance of these isolates in the pathogenesis of *Map* shouldn't be underestimated. The successful survival within ovine MDMs of the sheep isolates correlates well with epidemiological data and clinical evidence of virulence, as suggested by the capacity of sheep isolates to cause numerous outbreaks in sheep [7]. The successful survival phenotype of the bovine isolates within ovine MDMs correlated well with clinical evidence of virulence, as suggested by the capacity of bovine isolates to infect sheep in experimental conditions [32]. However, it should be pointed out that experimental infections typically involve high doses of *Map* and therefore may not accurately assess *Map* transmission in field conditions.

When the estimated log_10_ CFU numbers within ovine MDMs at 0 and 7 days p.i. were statistically analysed, the intracellular behaviour of the tested isolates varied depending on the time p. i. (*P*<0.001). Small increases in the estimated log_10_ CFU numbers from days 0 to 7 were observed for most of the isolates. In contrast, the genotype did not seem to significantly affect the behaviour of the selected isolates within ovine MDMs with types S, B and C showing similar survival phenotype after 7 days of infection (*P* = 0.635). A lack of correlation between genotype and intracellular behaviour of *Map* isolates was also previously observed in bovine macrophages [16]. Similarly, a lack of correlation between genotype and growth rate in the Bactec MGIT 960 system was observed for the ovine isolates; with C, S and B isolates from sheep growing at equivalent rates in MGIT cultures [27].

Previously, it was suggested that a bovine, a bison, and a human type-C isolates induced anti-inflammatory and antiapoptotic responses in bovine MDMs, which would favour bacterial survival and persistence [33]. In the current study, we showed that the successful survival of bovine and ovine isolates of *Map* within ovine MDMs correlated with an increased expression of BCL2-1, TIMP-1, TGFβ-1, and IL10. In addition, a reduced proinflammatory immune response mediated by IL2 and TNFα-2 was observed in the infected cells. Our results also demonstrated that ovine macrophages infected with ovine isolates of distinct genotype (C or S) did not differentially express BCL2-1, TIMP-1, TGFβ-1, IL10, IL2 and TNFα-2. Previously, the bovine isolate-6 of *Map* that grew within BoMac cells was reported to induce the expression of the anti-inflammatory cytokines TGFβ-1 and IL-10 in BoMac cells that antagonized the proinflammatory response by down-regulating the production of TNF-α which favour bacterial survival [16]. In regard to the apoptotic response, BoMac cells infected with the bovine isolate-6 had increased levels of expression of the apoptotic inhibitor BCL2-1 and of the inhibitor of tissue destruction TIMP-1 which might cause low levels of apoptosis. Overall, a strong correlation between the successful survival of *Map* isolates within bovine and ovine macrophages and the patterns of production of BCL2-1, TIMP-1, TGFβ-1, TNFα-2 and IL10 was observed. Our results also indicated that the proinflammatory cytokines IL1α and IL2 were down-regulated in BoMac cells and in ovine MDMs infected with bovine isolates of *Map,* respectively. It is well known that the expression of these cytokines in the presence of intracellular bacteria is one of the first steps leading to activation of macrophages and effective bacteria killing. Consistently with our results, other authors also observed an up-regulation of TGFβ and/or IL10 after the infection of bovine MDMs with live *Map* that down-regulated the production of TNFα [33]-[38]. Similarly, rapid intracellular macrophage growth rates by strains of *M. tuberculosis* strains correlated with rapid production of IL10 that antagonizes the proinflammatory response by down-regulating the production of TNFα in THP-1 cells during the early stages of infection [39].

Although MDMs can provide insights into *Map*-host interactions at the very early stages of the infection, this cellular model is unable to mimic later stages of the infection, such as early granuloma formation. To address this issue, three-dimensional *in vitro* models of granuloma have been recently developed. The formation of small, rounded granuloma-like structures was previously reported by coculture of human blood lymphocytes with autologous macrophages infected with live *M. tuberculosis*, *M. leprae*, or *M. bovis* or stimulation with mycobacterial antigens such as purified protein derivatives or lipomannan [21], [29], [40], [41]. In the present study, we reported for the first time the development of an *in vitro* model of ovine granuloma using *Map*-infected PBMCs cultured in an extracellular matrix composed of fibronectin and collagen, components of the surrounding tissue in which the natural granuloma is anchored. It was previously shown that the ability to develop a well-defined granulomatous response correlated with the severity of mycobacterial infections. When human PBMCs and macrophages were treated with 10^5^ heat-killed *M. tuberculosis* strain H37Rv they formed aggregates that remained very small and loose [21]. Similarly, human PBMCs infected with avirulent mycobacteria such as *M. smegmatis* and *M. avium* formed loose aggregates [20]. We observed that ovine PBMCs infected with *Map* isolates from sheep and cattle formed well defined aggregates after 10 days of incubation at 37 °C. *Map*-induced aggregates displayed morphological characteristics similar to natural granulomas, such as three-dimensional aggregation of lymphocytes around macrophages In accordance with our results, granulomatous lesions consistent with *Map* infection have been recently found in tissue sections from lambs experimentally infected with bovine (C-type) and ovine (S-type) isolates of *Map* [42]. The advantages of *in vitro* models of granuloma include reduced cost, increased control, and that they can provide insights into host-mycobacteria interactions at stages of granuloma formation too early to address with animal models. However, the granuloma constitutes a complex immune microenvironment highly affected by additional physiological signals (ie. growth factors and cytokines) which are exclusively produced in infected tissues. As consequence, certain aspects of *in vivo* granulomas may be different or absent in *in vitro* models, including intra-granulomatous necrosis, accumulation of fibrin and collagen, and presence and distribution of bacilli. Three-dimensional *in vitro* models of granuloma may be very useful to: (i) understand what factors or molecules play a role in granuloma formation and in its continued integrity, (ii) evaluate the granuloma-inducing activity of particular antigens or attenuated mutants, and (iii) provide a platform for testing vaccine and drug candidates.

In order to protect paratuberculosis-free herds, control programs have been developed in some countries. The success of these control programs depends on the ability to make decisions regarding on-farm management practices and the movement of animals between regions. For instance, policies regarding mixed farming of cattle and sheep have been based on the apparent host specificity of *Map*. However, our results suggested that sheep might be susceptible to infection with *Map* isolates not only from sheep but from cattle, goats, deer, fallow deer and wild boar as well. Therefore, the implementation of measures to prevent the risk of *Map* transmission from these animal species to sheep in multispecies livestock operations or on farms where sheep share pastures with wildlife animal should be recommended. Because a lack of correlation between genotype and intracellular phenotype of *Map* isolates in ovine macrophages was observed, destocking polices that aim to eliminate *Map* should assume equivalence of strains and should not be based on genotype distinction.

## Conclusion

*Map* isolates from cattle, sheep, goats, fallow deer, deer and wild boar showed successful survival phenotype within ovine macrophages regardless of genotype. This phenotype correlated with stimulation of anti-inflammatory, antiapoptotic and antidestructive responses within ovine macrophages. In addition, we showed that two *Map* isolates from cattle and sheep with distinct genotypes (C and S) were able to induce the formation of *in vitro* granulomas with a well-defined edge and comparable to the granulomas observed in clinical specimens with respect to the cellular components involved. All together our findings reinforce the hypothesis that *Map* isolates from cattle, goats, deer, and wild boar may have similar clinical consequences in sheep than sheep isolates of *Map* which according to our results are not expected to have a selective advantage in causing ovine paratuberculosis.

## Acknowledgments

Samples from fallow deer were provided by Dr. Prieto from the Department of Agriculture of the Regional Government of the Principality of Asturias (Spain). Samples from wild-boar and deer were provided by the Instituto de Investigación en Recursos Cinegéticos (IREC). Samples from India, Portugal and USA were kindly provided by S. V. Singh, A.C. Coelho and R. Whitlock, respectively. We thank Dr. Esmeralda Minguijon and Nieves Gomez at the Department of Histopathology (NEIKER-Tecnalia) for excellent technical assistance. Technical and human support provided by SGIker (UPV/EHU, MINECO, GV/EJ, ERDF and ESF) is gratefully acknowledged. We thank Dr. Luiz Bermudez from Oregon State University for helpful discussions. We are grateful to Kyle Hearn and Liam Fitzgerald for the careful editing of the manuscript.
